# Breast cancer robotic nipple sparing mastectomy: evaluation of several surgical procedures and learning curve

**DOI:** 10.1186/s12957-019-1567-y

**Published:** 2019-02-06

**Authors:** G. Houvenaeghel, M. Bannier, S. Rua, J. Barrou, M. Heinemann, A. Van Troy, E. Lambaudie, M. Cohen

**Affiliations:** Department of Surgical Oncology, Institut Paoli Calmettes and CRCM and Aix-Marseille Université, 232 Bd de Sainte Marguerite, 13009 Marseille, France

**Keywords:** Robotic surgery, Breast cancer, Nipple sparing mastectomy

## Abstract

**Background:**

Few studies of robotic nipple sparing mastectomy (NSM) were reported. We report feasibility of robotic NSM and determine standard surgical procedure and learning curve threefold.

**Methods:**

A cohort of patients with robotic NSM for breast cancer was analyzed. Complications and post-operative hospitalization stay were reported. The same technic was used for all patients except for skin and nipple areolar complex (NAC) dissection. Differences between three surgical procedures of NAC dissection were analyzed: group 1, dissection with robotic scissors using coagulation; group 2, dissection with robotic scissors without coagulation; and group 3, dissection with non-robotic scissors and then robotic dissection. We explored possible effect of learning curve among patients from group 1 with the same surgical procedure.

**Results:**

Twenty-seven NSM with immediate breast reconstruction for breast cancers, 22 invasive and 5 in situ, were performed, with robotic latissimus dorsi-flap (RLDF) only in 17 cases, RLDF and breast implant in 6 cases, and implant alone in 4 cases. Repartition according to 3 surgical procedure groups was 16, 5, and 6 patients. Mean time of surgery and anesthesia decrease according to groups 1 to 3. Among 16 patients from group 1, time of surgery and anesthesia decreased with learning curve. Post-operative hospitalization decreased from group 1 to 3. We reported a total of 11 complications, with significant difference between groups (10 for group 1). Skin complications were higher for group 1 in comparison with groups 2–3 (*p* = 0.02).

**Conclusion:**

Robotic NSM can be performed with a brief learning. Standardized technique is proposed with non-robotic scissors superficial dissection and then dissection with robot.

## Introduction

Robotic oncologic surgery is considered a valid endoscopic technique for several indications including urologic, colorectal, and gynecologic surgery. Nipple sparing mastectomy (NSM) is today considered as a valid procedure for prophylactic mastectomy and an acceptable option for breast cancer (BC) therapeutic mastectomy [1–5]. Very few studies of robotic mastectomy were reported [6–9], and some studies were specifically published about endoscopic robotic latissimus dorsi-flap dissection [10–18].

Since 2007, we have now a strong experience of gynecologic oncologic robotic surgery not only for hysterectomy but also for more complex procedures [19–22]. These experiences conduct us to start breast robotic surgery development.

The aim of this study was to report feasibility of robotic NSM and determine standard surgical procedure and learning curve threefold.

## Methods

### Patients

Robotic mastectomies (RM) and immediate breast reconstruction (IBR) were performed by one surgeon during 16 months (from the first procedure in November 2016 to February 2018). All patients were informed of robotic assistance surgery. Our institutional ethical committee approved robotic breast surgery procedures.

We determined characteristics of patients (age, body mass index (BMI), tobacco use, diabetes, ASA status, breast volume), previous treatment for breast cancer (BC) (sentinel lymph node biopsy, axillary lymph node dissection (ALND), neo-adjuvant chemotherapy, previous breast radiotherapy), indications of NSM (primitive BC or local recurrence, reconstruction with robotic latissimus dorsi-flap (RLDF), and or breast implant).

Surgical technic with type of Da Vinci system, number of trocars, skin incision, duration of anesthesia, and surgery were reported according to period of treatment and association of surgical procedures (mastectomy, breast implant, RLDF, ALND, and contra lateral breast surgery). Six chronologically periods of 3 months was determined.

Complication rate was determined with Clavien-Dindo grading [23]. Re-operation rate, type of complication, and number of post-operative hospitalization days were analyzed.

### Groups of surgical procedures

The same technic was used for all patients except for skin and nipple areolar complex (NAC) dissection, determining three groups: group 1, dissection with robotic scissors using coagulation; group 2, dissection with robotic scissors without coagulation; and group 3, dissection with non-robotic scissors after subcutaneous infiltration with adrenaline serum and then robotic dissection.

We explored possible effect of learning curve among patients from group 1 with the same surgical procedure (NSM and robotic LDF with or without breast implant).

### Statistics

Main characteristics were reported with median, mean, and confidence interval 95% (CI 95%) for quantitative criteria. Comparisons were performed using *χ²*, *t* test, and binary logistic regression with SPSS 16.0.

## Results

Characteristics of patients are reported in Tables 1 and 2: 27 NSM with immediate breast reconstruction (IBR) for 10 (37%) local recurrences (7 invasive and 3 DCIS) and 17 (63%) primitive BC (15 invasive and 2 DCIS) were performed. Breast reconstruction used autologous RLDF only in 17 cases, RLDF and breast implant in 6 cases, and breast implant alone in 4 cases. Mean implant sizes were respectively 390 cc (range 311–490) for RLDF with implant and 283 (range 230–330) for breast implant reconstruction alone. Distribution according to 3 surgical procedure groups was 16, 5, and 6 patients.

**Table 1:** Characteristics of patients

		Group 1		Group 2		Group 3		Total		χ²
		Nb	%	Nb	%	Nb	%	Nb	%	p
Type reconstruction	Implant	1	6.2	2	40.0	1	16.7	4	14.8	0.15
	Implant + RLDF	5	31.2	1	20.0	0		6	22.2	
	RLDF	10	62.6	2	40.0	5	83.3	17	63.0	
Breast cancer	Primitive	10	62.5	3	60.0	4	66.7	17	63.0	0.973
	Recurrence	6	37.5	2	40.0	2	33.3	10	37.0	
Cup size	A–B	8	50.0	2	18.2	4	66.6	14	51.8	0.466
	C	6	37.5	3	60.0	2	33.3	11	40.7	
	>C	2	12.5	0		0		2	7.4	
Tobacco	No	12	75.0	3	60.0	5	83.3	20	74.1	0.673
	Yes	4	25.0	2	40.0	1	16.7	7	25.9	
Diabetes	No	15	93.8	5	100	6	100	26	96.3	0.700
	Yes	1	6.2	0		0		1	3.7	
ASA	1	8	50.0	2	40.0	2	33.3	12	44.4	0.763
	2	8	50.0	3	60.0	4	66.7	15	55.6	
Previous contralateral BC	No	12	75.0	4	80.0	4	66.7	20	74.1	0.874
	Yes	4	25.0	1	20.0	2	33.3	7	25.9	
Previous conservative breast surgery	No	8	50.0	3	60.0	2	33.3	13	48.1	0.660
	Yes	8	50.0	2	40.0	4	66.7	14	51.9	
Previous SLNB	No	13	81.2	3	75.0	2	33.3	19	70.4	0.092
	Yes	3	18.8	1	25.0	4	66.7	8	29.6	
Previous ALND	No	11	68.8	3	60.0	6	100	20	74.1	0.240
	Yes	5	31.2	2	40.0	0		7	25.9	
Previous homolateral Radiotherapy	No	9	56.2	3	60.0	4	66.7	16	59.3	0.906
	Yes	7	43.8	2	40.0	2	33.3	11	40.7	
Neo-adjuvant chemotherapy	No	13	81.2	5	100	6	100	24	88.9	0.3013
	Yes	3	18.8	0		0		3	11.1	
Implant volume	< = 300	3	50.1	1	33.3	0		4	40.0	0.65
	311–330	2	33.4	1	33.3	1	100	4	40.0	
	> 400	1	16.7	1	33.3	0		2	20.0	
Axillary surgery	No	10		3		4		17	62.9	0.90
	SLNB	5		2		2		9	33.3	
	ALND	1		0		0		1	3.7	
Trocar number	2	16	100	3	60.0	6	100	25	92.6	0.009
	3	0		2	40.0	0		2	7.4	
Da Vinci system	SI	11	68.8	2	40.0	1	16.7	14	51.9	0.079
	XI	5	31.2	3	60.0	5	83.3	13	48.1	
Procedure surgical number	1	1	6.2	2	40.0	1	16.7	4	14.8	0.057
	2	4	25.0	2	40.0	5	83.3	11	55.6	
	3	8	50.0	1	20.0	0		9	33.3	
	4	3	18.8	0		0		3	11.1	
Post-operative hospitalization	< 4 days	7	43.8	3	60.0	4	66.7	14	51.9	0.582
	> = 4 days	9	56.2	2	40.0	2	33.3	13	48.1	
BMI	< 23.5							22		0.763
	> = 23.5							5		
Surgical time	< 360	8	50.0	3	60.0	5	83.3	16	59.3	0.366
	> = 360	8	50.0	2	40.0	1	16.7	11	40.7	

*Abbreviations*: *RLDF* robotic latissimus dorsi flap, *SLNB* sentinel lymph node dissection, *ALND* axillary lymph node dissection, *BMI* body mass index

**Table 2:** Characteristics of patients and surgery according to three groups

		Group 1	Group 2	Group 3	Total
Age	Median	49.5	57.0	50.0	51
	Mean	52.4	51.0	45.3	51.2
	CI 95%	45.9–58.9	29.8–72.2	14–76.8	45.75–56.67
Weight	Median	60.5	57.0	58.0	59
	Mean	59.1	62.8	58.3	59.79
	CI 95%	55.6–62.6	41.0–84.6	44.6–72.0	55.87–63.72
Patient size	Median	164.5	164	168	164.5
	Mean	163.8	164.6	166	164.25
	CI 95%	160.1–167.5	160.2–169.0	152.9–179.1	161.7–166.8
BMI	Median	22.25	21.19	20.7	21.58
	Mean	22.0	23.0	21.1	22.11
	CI 95%	21.0–23.0	16.4–29.7	17.8–24.5	20.9–23.3
Anesthesia time	Median	448.5	349	375	431
	Mean	436.6	393.2	344.3	416
	CI 95%	394–479	242–544	99.3–589	376.7–455.4
Surgical time	Median	370.5	265	285	349
	Mean	372.5	303.4	257.7	343.75
	CI 95%	330–415	167–439	24–491	304.0–383.5
Post-operative hospitalization	Median	4	3.0	2.5	3.5
	Mean	3.88	2.8	2.75	3.5
	CI 95%	3.1–4.65	1.2–4.4	1.23–4.27	2.94–4.14
Mastectomy weight	Median	237.5	194	350.5	237.5
	Mean	303	250	351.5	288.9
	CI 95%	227–378	23.5–477	65.4–638	228–350
Surgical time NSM + RLDF	Median	390	360	335	351
	Mean	374	364	324	362
	CI 95%	329–419	114–613	280–368	329–395
Anesthesia time NSM + RLDF	Median	455	457	408	442
	Mean	440	460	413	437
	CI 95%	395–485	179–741	362–464	403–470

*Abbreviations*: *BMI* body mass index, *NSM* nipple sparing mastectomy, *RLDF* robotic latissimus dorsi flap

Robotic mastectomies were performed in 14 patients with SI Da Vinci (51.9%) and 13 with XI system (XI was available since 24 February 2017: 5 with SI and 14 with XI (73.7%).

### Surgical procedure

When mastectomy was performed with a concomitant RLDF, patients’ installation were dorsal decubitus then in side decubitus. For robotic NSM, installation was realized with anteflexion of arm in order to use robotic arm without strong limitation.

A vertical axillar incision, about 4 to 6 cm according to breast volume, on anterior axillary line allowed the beginning of the dissection on 3–4 cm for subcutaneous plan and a limited dissection under incision along anterior axillary line in order to introduce one robotic trocar about 6 cm under axillar incision. Then, a GelPoint mono-trocar was introduced through the axillar incision with two robotic trocars (one trocar for a 0° camera (Intuitive Surgical, Denzlingen, Germany) and one trocar for AirSeal insufflation also used for the assistant surgeon when necessary. We used a low pressure (7 mm). We introduced a monopolar scissors and bipolar clamp into up and down robotic trocars with camera in the middle robotic trocar. After superficial dissection, we started the dissection between major pectoralis muscle and breast gland, then we realized section of gland periphery on upper, internal and lower quadrant with robotic scissors using monopolar coagulation. After mono-trocar removal, we systematically performed a retro NAC biopsy with NAC eversion without extemporaneous analysis and verification of complete gland removal. We performed a complete resection of tissue under NAC and particularly under the nipple with only preservation of skin NAC.

After mastectomy, we start by the same incision and mono-trocar RLDF dissection with patient installation in lateral side. After mobilization of LDF, fixation of muscle was performed with several sutures and aspirate drainage disposed in dorsal area (2 drains through the inferior incision for robotic trocar) and in mastectomy area (1 drain). We do not perform dorsal padding. When implant was associated with RLDF, implant was disposed under the muscle without changing the patient’s position (patient installation in lateral side). When only implant was used, a robotic dissection of major pectoralis muscle provide pocket to manually introduce the prosthesis.

### Time of surgery and anesthesia

**Fig. 1:**
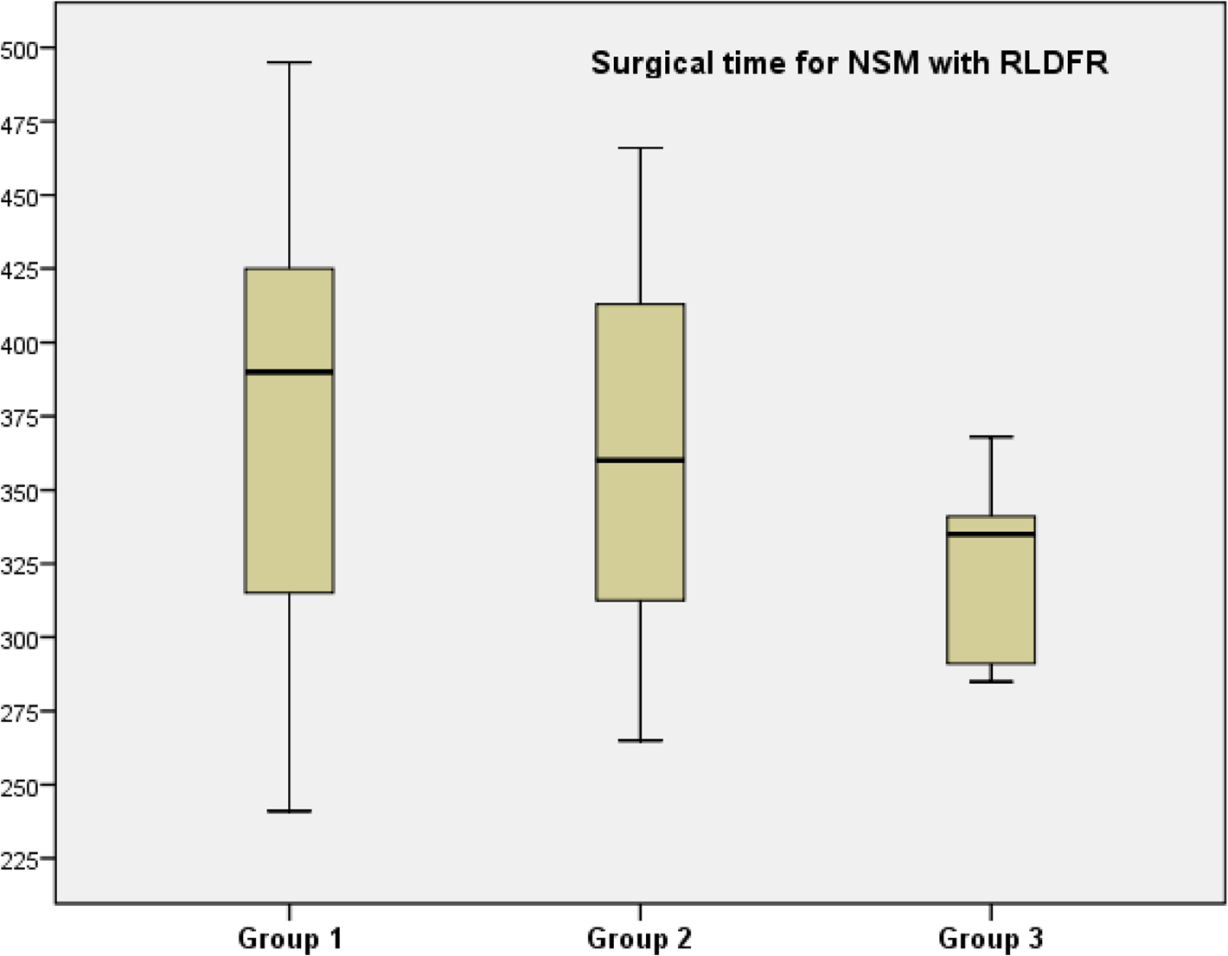
Surgical time according to three groups for patients with NSM and RLDFR

**Fig. 2:**
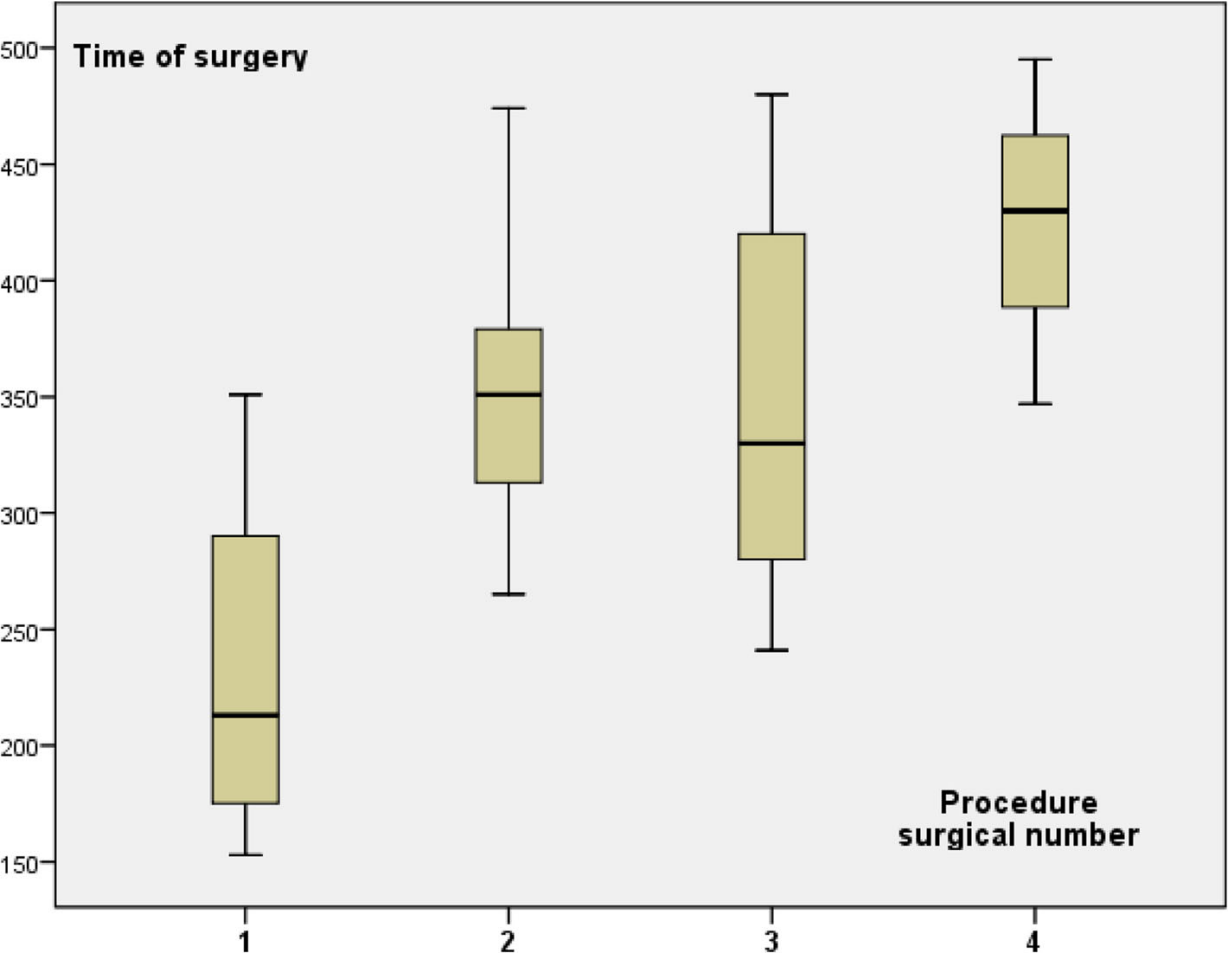
Time of surgery according to procedure surgical number (NSM, RLDFR, implant, ALND)

Time of anesthesia is recorded from anesthesia induction to tracheal extubation. Time of surgery include all procedures and several installations from skin incision to the end of skin suture. Mean time of surgery decrease according to groups 1 to 3 (Table 2) with higher difference when we analyzed only NSM with concomitant RLDFR (Table 2 and Fig. 1). These differences were not significant in relation with the small number of patients in each group. Time of surgery increased according to association of procedures number (Fig. 2). Mean time of anesthesia also decrease according to groups 1 to 3 (Table 2) with higher difference when we analyzed only NSM with concomitant RLDF (Table 2).

Time of surgery and anesthesia were different between procedures performed with SI or XI system (Table 3), but XI system was used only from 24 February 2017. Median time of surgery and anesthesia for 5 SI vs 14 XI procedures since 24 February 2017 were not significantly different, respectively 347mn and 300mn (mean 316 and 312; *E* 153–456 and 197–420) for surgery and 455 and 380 (mean 412 and 384; *E* 234–575 and 288–480) for anesthesia (*t* test 0.936).

Specific times of surgery for NSM were different according to groups (means 161mn, 184mn, and 117mn respectively for groups 1 to 3, with no difference between groups 1 and 2 and significant differences between group 2 and 3 (*p* = 0.003) and group 1 and 3 (*p* = 0.010)) and according to breast cup size A–B vs C–D (mean 138mn vs 177mn; *p* = 0.018). There was no significant difference of cup sizes between the three groups.

### Learning curve

**Fig. 3:**
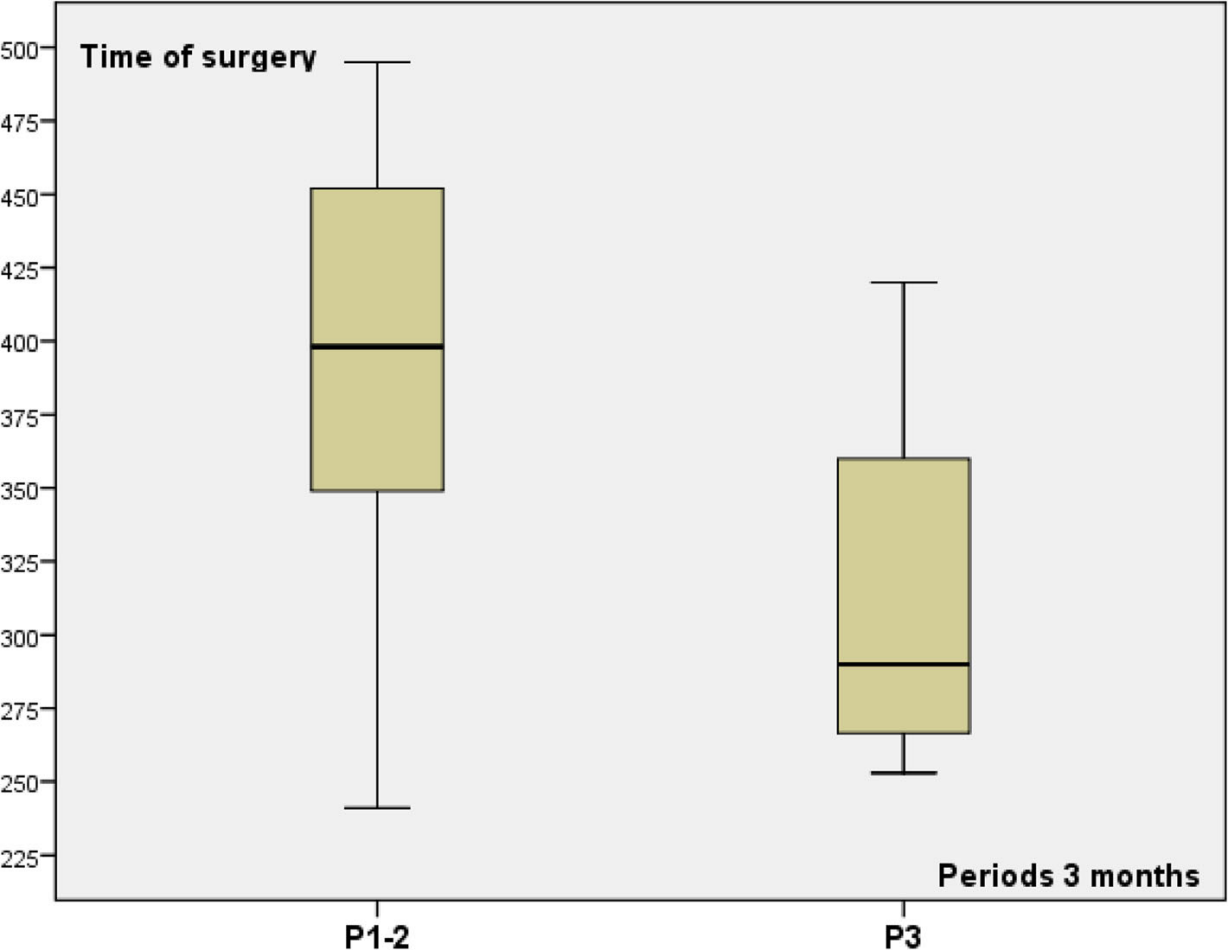
Time of surgery for group 1 patients according to periods of 3 months

**Fig. 4:**
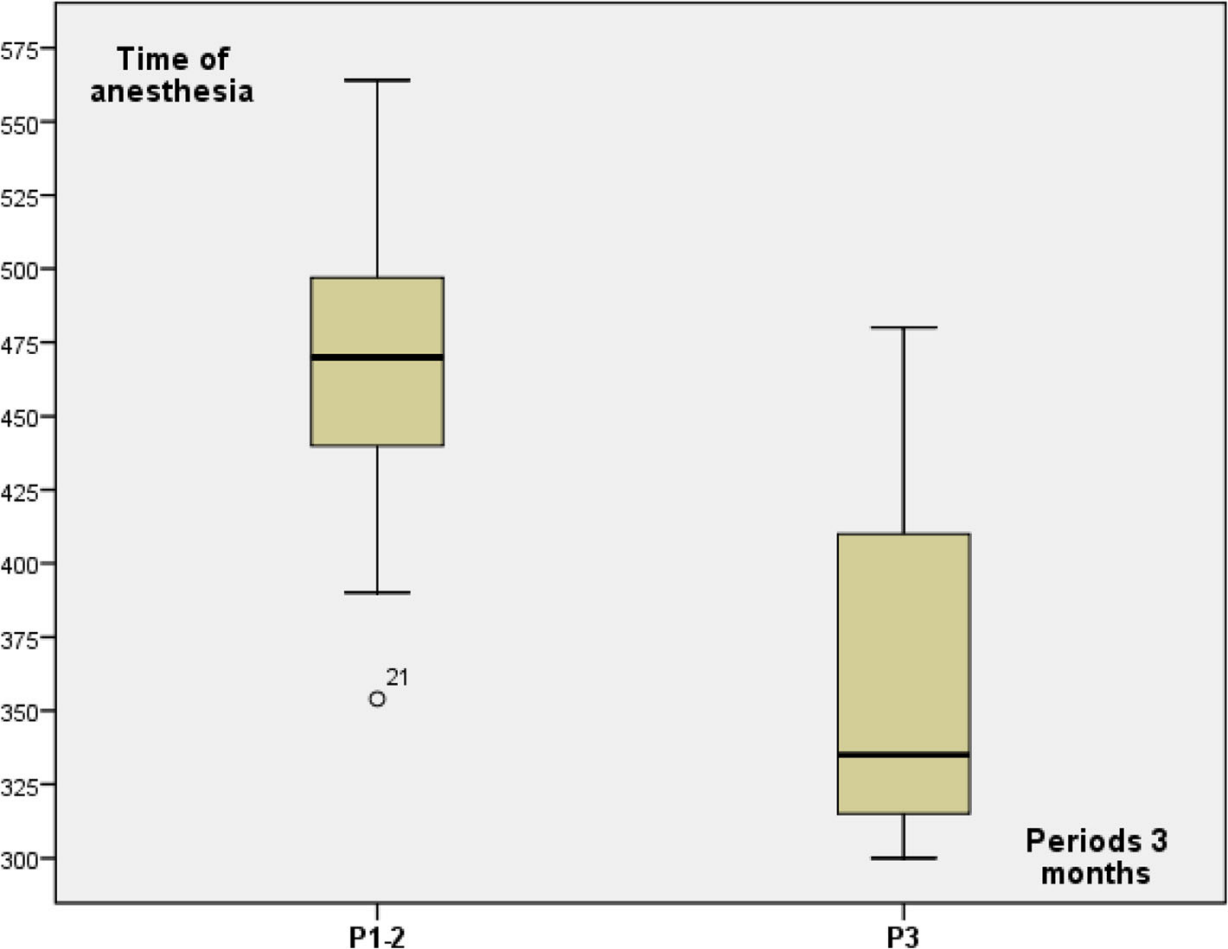
Time of anesthesia for group 1 patients according to periods of 3 months

Among 15 patients from group 1 with the same surgical procedure (NSM and RLDF), 3 periods of 3 months was determined (P1 to P3) with respectively 3, 8, and 4 patients in order to explore learning curve impact. Time of surgery and anesthesia decreased during third period (P3) in comparison with the two first periods (P1–2) (Table 3 and Figs. 3 and 4). Time of surgery and anesthesia were lesser for P3 in comparison with P1–2 (Table 3) without difference between P1 and P2. Number of associated surgical procedures was 2 or 3 for patients operated during P1–2 (three patients with three procedures) and three procedures for four patients operated during P3.

### Outcome

Post-operative hospitalization decrease from group 1 to 3 (median days respectively 4, 3, and 2.5 (Table 2). We reported a total of 11 complications, with significant difference between groups (Table 4). Ten of these complications were observed in group 1. Seven complications of NSM were Clavien-Dindo grade II or III (7/27: 25.9%). Types of complications are reported in Table 4. The more important rate was in relation with dorsal lymphocele. Skin necrosis, about 2 cm out of NAC (1 patient: grade II) and cutaneous small blistering (5 patients: grade I) was significantly higher for group 1 in comparison with groups 2 and 3 (6/16 vs 0/11; *p* = 0.02). Re-operation was necessary for four patients within three cases explantation of prosthesis. For the last patient, a conversion from robotic to open surgery was required after half of dissection for bleeding on arterial perforant of arterial internal mammary, but without post-operative complication. Patients with Clavien-Dindo grade II–III complications and their characteristics are reported on Table 5.

**Table 3:** Time of surgery and anesthesia

	Surgical time				t test	Anesthesia time				t test
	Median	Mean	CI 95%	Range	p	Median	Mean	CI 95%	Range	p
SI	375	371	312–429	153–495	0.086	463.5	447	391–503	234–575	0.059
XI	300	312	273–351	197–420		380	384	343–424	288–480	
P1	390	400	212–587	330–480		514	489	267–712	390–564	
P2	409	394	327–462	241–495		462.5	460	412–509	354–563	
P3	290	313	196–430	253–420		335	362.5	235–490	300–480	
P1–2	398	396	345–447	241–495	0.083	470	468	425–511	354–564	0.020
P3	290	313	196–430	253–420		335	362	235–490	300–480	
2 SP	405	409	326–491	351–474		497	499	414–583	438–563	
3 SP	315	338	266–410	241–480		372	400	325–475	300–564	
4 SP	430	424	240–608	347–495		470	468	437–499	455–480	

*Abbreviations*: *SI* Da Vinci SI system, *XI* Da Vinci XI system, *P1–2–3* periods 1–2–3, *SP* surgical procedures

**Table 4:** Complication results

		Group 1		Group 2		Group 3		Total	
		Nb	%	Nb	%	Nb	%	Nb	%
Complications		10	62.5	1	20.0	0		11	40.7
Conversion		0		0		1	16.7	1	3.7
Re-operation		3	18.8	1	20.0	0		4	14.8
Clavien-Dindo Mastectomy	0	6	37.5	4	80.0	6	100	16	59.3
	1	4	25.0	0		0		4	14.8
	2	3	18.8	0		0		3	11.1
	3	3	18.8	1	20.0	0		4	14.8
Type complication	Infection	3	18.75	1	20.0	0		4	14.8
	Hematoma	2	12.5	1	20.0	0		3	11.1
	Skin necrosis	1	6.25	0		0		1	3.7
	Lymphocele	9	56.25	1	20.0	0		10	37.0
	Explantation implant	2	12.5	1	20.0	0		3	30.0*
	Skin blistering	5	31.25	0		0		5	18.5

*3/10 breast implant

**Table 5:** Characteristics of seven patients with grade II–III complications

	Re-operation (grade III)				Grade II complication		
Group	1	1	1	2	1	1	1
Previous radiotherapy	No	No	Yes	No	No	No	No
Neo-adjuvant chemotherapy	Yes	No	Yes	No	No	No	No
BMI	23.80	20.96	21.10	32.18	20.32	22.68	18.14
Weight mastectomy	500	244	231	555	160	212	348
Implant	Yes	No	No	Yes	No	Yes	No
Time of surgery	430	474	280	466	330	241	420
Time of anesthesia	480	563	300	575	390	354	480
Surgical procedure number	4	2	3	3	3	3	3
RLDFR	Yes	Yes	Yes	Yes	Yes	Yes	Yes
ALND	Yes	No	No	No	No	No	No
Cup size	D	C	C	C	B	B	C
Re-operation	Yes	Yes	Yes	Yes	No	No	No

*Abbreviations*: *BMI* body mass index, *RLDFR* robotic latissimus dorsi-flap reconstruction, *ALND* axillary lymph node dissection

In univariate analysis, complication rate was correlated with group 1 vs group 2–3 (10/16 vs 1/11: *p* = 0.007), group 1–2 vs group 3 (11/21 vs 0/6: 0.027), associated surgical procedure number 1 or 2 vs 3 or 4 (2/15 vs 9/12: 0.002), 6 periods of 3 months (0.006), and we observed a significant borderline result for BMI < or > = 23.5 (7/22 vs 4/5: 0.071). The following criteria were non-significant: time of surgery < or > = 360 (6/16 vs 5/11: 0.492), time of anesthesia < or > = 382 mn (4/10 vs 7/17: 0.637), type of system SI vs XI (7/14 vs 4/13: 0.267), previous neo-adjuvant chemotherapy or not (2/3 vs 9/24: 0.357), previous ipsilateral radiotherapy or not (5/11 vs 6/16: 0.492), previous ipsilateral conservative surgery or not (5/14 vs 6/13: 0.436), NSM for primitive BC or local recurrence (7/17 vs 4/10: 0.637), ASA 1 or 2 (6/12 vs 5/15: 0.315), implant or not (4/10 vs 2/17: 0.9), and cup size breast A–B or C–D (5/14 vs 6/13:0.436).

In univariate analysis, re-operation rate was correlated with previous neo-adjuvant chemotherapy or not (2/3 vs 2/24: 0.049) (OR 22.0, CI 95% 1.33–362, *p* = 0.031) and cup size breast A–B or C–D (0/14 vs 4/13: 0.041). Other criteria were non-significant: group 1 vs group 2–3 (3/16 vs 1/11: 0.455), group 1–2 vs group 3 (4/21 vs 0/6: 0.341), time of surgery < or > = 360 (1/16 vs 3/11: 0.169), time of anesthesia < or > = 382mn (1/10 vs 3/17: 0.523), BMI < or > = 23.5 (2/22 vs 2/5: 0.144), surgical procedure number 1–2 vs 3–4 (1/15 vs 3/12: 0.216), 6 periods of 3 months (0.347), type of system SI vs XI (3/14 vs 1/13: 0.327), previous ipsilateral radiotherapy or not (1/11 vs 3/16: 0.455), previous ipsilateral conservative surgery or not (1/14 vs 3/13: 0.269), NSM for primitive BC or local recurrence (4/17 vs 0/10: 0.136), ASA 1 or 2 (1/12 vs 3/15: 0.389), and implant or not (2/10 vs 7/17: 0.28). In binary logistic regression, any factors remain significantly correlated to re-operation rate.

In univariate analysis, complications grade II–III rates were correlated with associated surgical procedure number 1–2 vs 3–4 (1/15 vs 6/12: 0.016), previous homo-lateral conservative surgery or not (1/14 vs 6/13: 0.029), and NSM for primitive BC or local recurrence (7/17 vs 0/10: 0.022). Other criteria were non-significant: time of surgery < or > = 360 (3/16 vs 4/11: 0.279), BMI < or > = 23.5 (5/22 vs 2/5: 0.388), time of anesthesia < or > = 382mn (2/10 vs 5/17: 0.475), 6 periods of 3 months (0.215), type of system SI vs XI (5/14 vs 2/13: 0.224), implant or not (3/10 vs 4/17:0.60), previous neo-adjuvant chemotherapy or not (2/3 vs 5/24: 0.156), previous homo-lateral radiotherapy or not (1/11 vs 6/16: 0.112), ASA 1 or 2 (4/12 vs 3/15: 0.364), cup size breast A–B or C–D (2/14 vs 5/13: 0.161), group 1 vs group 2–3 (6/16 vs 1/11: *p* = 0.112), group 1–2 vs group 3 (7/21 vs 0/6: 0.131), and time of surgery < or > = 360 (3/16 vs 4/11: 0.279). In binary logistic regression, including NSM for primitive BC or local recurrence and associated surgical procedure number 1–2 vs 3–4 (with exclusion of previous ipsilateral conservative surgery or not which is strongly correlated with NSM for primitive BC or local recurrence), complications grade II–III were correlated with number of associated surgical procedures which remain highly significant (OR 54.0, CI 95% 2.8–1040, *p* = 0.008) but indication of NSM was not significant (0.998).

### Breast cancer treatment

Previous radiotherapy was observed in 10 patients with local recurrences and for 1 patient with neo-adjuvant chemotherapy and radiotherapy. Neo-adjuvant chemotherapy was performed in three cases. Trastuzumab was administered in two cases.

## Discussion

We reported our experience with robotic NSM for BC with evaluation of three surgical technics to perform mastectomy in order to propose a standardized procedure corresponding to the best and quicker procedure. Among 15 patients of group 1 using the same technic (dissection with robotic scissors with monopolar coagulation), we have evaluated the learning curve according to three periods of 3 months on surgical and anesthesia time. The main conclusions are (1) the third procedure of mastectomy dissection appeared to be the safer and quicker procedure (dissection with non-robotic scissors after subcutaneous infiltration and then robotic dissection), (2) learning curve need approximatively 10–11 robotic mastectomies even for surgeon with previous experience of robotic surgery for others indications.

This surgical procedure with incision in axillary basin allowed good cosmetic results without scar on breast and without dorsal scar with RLDF. IBR allows better quality of life in comparison with mastectomy without reconstruction [24], and small axillary incision with NSM increase good cosmetic results and contribute significantly to a woman’s body image and quality of life [25, 26]. These robotic NSM were realized without difference between SI and XI system, but XI system is easier for dissection with easier mobilization of robotic arms without conflictual movement between robotic arms and patient arm. Moreover, when RLDF is planned, quicker and easier re-installation was observed with XI system. We had used more often XI system since the installation of the second Da Vinci robot.

This study is the first specifically dedicated to BC and the first study with evaluation of several surgical dissections technics. Toesca et al. reported [9] a case series of 24 consecutive patients (29 procedures) for 10 prophylactic surgeries and 19 BC performed to access feasibility, reproducibility, and safety. A stronger selection of patients was reported in his study in comparison with our study. All patients in Toesca et al.’s study [9] had no associated comorbidities, a BMI < 25, were classified as low risk for anesthesia, small breast volume (weight of specimen range between 200 and 300 g) without ptosis > 2, without diabetes, and without previous radiotherapy. For comparison, we have included only BC patients: 11 with previous radiotherapy, 1 with BMI > =25, 13 (48.1%) with breast cup size C–D, 12 (44.4%) with mastectomy weight > 300 g, and 6 breast implant volume > 300 cc (60%). Greater breast volume observed in our study can explain longer incision for NSM in comparison with Sarfati et al.’s and Toesca et al.’s studies [8, 9]. Moreover, we have performed concomitant RLDF in 85.2% of patients in comparison with implant breast reconstruction for all patients in Toesca et al.’s study [9]. Sarfati et al. [8] reported one case of robotic mastectomy with XI system including two prophylactic mastectomies with implant reconstruction disposed in subcutaneous position for a patient with breast cup size C.

We observed some surgical differences with these previous studies. Like Toesca et al., we used a mono-trocar system with the same small previous dissection before robot docking. However, the second operative robotic arm was disposed out of mono-trocar on anterior axillary line 6 cm under axillary incision. Sarfati et al. [8] do not use mono-trocar. For superficial gland dissection, we propose to perform this time of surgery with non-robotic scissors without coagulation (bipolar coagulation was performed after docking and insufflation) and retro NAC biopsy with more than 2 cm at clinical and radiological exam for indication of NAC preservation [4, 27]. Toesca et al. [9] reported two cases of NAC removal for involvement of NAC by invasive carcinoma or DCIS (2/19: 10.5%) with proposition of NSM for patients with tumor-nipple distance greater than 1 cm. Safety of NAC preservation for BC have been well documented with mainly discussions about tumor distance and about thickness not removed under the skin and NAC [28–30]. NSM can be proposed in selected cases of recurrent BC (37% of our cases) [31], after neo-adjuvant chemotherapy with or without neo-adjuvant radiotherapy (11.1% of our cases) [32–34], and for DCIS (18.5% of our cases) [35].

Duration of surgery reduced gradually for the first case to the final cases with a total length of time of 7 h for the first robotic surgery to around 3 h for the last cases in Toesca et al.’s study for NSM with breast implant reconstruction [9]. Our median surgical times for NSM with RLDF decrease from 370 mn to 285 mn for the third group and for specific time of NSM from 161 to 117 mn. Conversion rates were 6.9% (2/29) for Toesca et al. [9] and 3.7% in our study (1/27). Small blistering was reported in two patients (2/24: 8.3%) by Toesca et al. [9]; we observed more skin breast complications with one small skin necrosis and five small skin blistering, all of them in group 1. Clavien-Dindo grade II–III complications were more often observed in our study for group 1 procedures with no such complication in group 3 and were correlated in logistic regression with surgical procedure number higher than 2.

Robotic surgery is usually considered as a very expensive procedure. Fixed costs (maintenance and amortization) and cost of robotic instruments can provide more costs than non-robotic endoscopy or open surgery. Additional costs per procedure were low when about 300 procedures were performed with 1 Da Vinci system [36] and minimized by use of only 2 robotic arms for dissection. A short learning curve would also no doubt decrease the operating theater costs [36].

## Conclusion

The technique of robotic NSM can be achieved with a short learning curve for surgeons with previous experience of robotic surgery. Standardized technique proposed consisted to perform superficial gland dissection with non-robotic scissors and then to perform all other dissection with robot through a mono-trocar insert in axillary small incision. Reconstruction can be performed with breast implant after robotic dissection of major pectoralis muscle or with RLDF dissected by the same axillary incision with or without implant. Complications grade II–III are correlated with more than two surgical procedures including NSM, RLDF, implant, and ALND. A prospective evaluation is necessary and planned in order to confirm these results and determine advantages over an open approach. To increase breast reconstruction volume autologous fat grafting is usually performed after this procedure.

## References

[1] Smith BL, Tang R, Rai U, Plichta JK, Colwell AS, Gadd MA (2017). Oncologic safety of nipple-sparing mastectomy in women with breast cancer. J Am Coll Surg.

[2] Li M, Chen K, Liu F, Su F, Li S, Zhu L (2017). Nipple sparing mastectomy in breast cancer patients and long-term survival outcomes: an analysis of the SEER database. PLoS One.

[3] Muller T, Baratte A, Bruant-Rodier C, Bodin F, Mathelin C. Oncological safety of nipple-sparing prophylactic mastectomy: a review of the literature on 3716 cases. Ann Chir Plast Esthet. 2018;63(3):e6-e13. 10.1016/j.anplas.2017.09.005 29030030

[4] Galimberti V, Vicini E, Corso G, Morigi C, Fontana S, Sacchini V, Veronesi P (2017). Nipple-sparing and skin-sparing mastectomy: review of aims, oncological safety and contraindications. Breast.

[5] Munhoz AM (2017). Outcome evaluation after 2023 nipple-sparing mastectomies: our experience. Plast Reconstr Surg.

[6] Toesca A, Peradze N, Galimberti V, Intra M, Gentilini O, Sances D (2017). Robotic nipple-sparing mastectomy and immediate breast reconstruction with implant: first report of surgical technique. Ann Surg.

[7] Toesca A, Manconi A, Peradze N, Loschi P, Panzeri R, Granata M (2015). Preliminary report of robotic nipple-sparing mastectomy and immediate breast reconstruction with implant. Eur J Cancer.

[8] Sarfati B, Honart J-F, Leymarie N, Rimareix F, Khashnam HA, Kolb F (2017). Robotic da Vinci Xi-assisted nipple-sparing mastectomy: first clinical report. Breast J.

[9] Toesca A, Peradze N, Manconi A, Galimberti V, Intra M, Colleoni M (2017). Robotic nipple-sparing mastectomy for the treatment of breast cancer: feasibility and safety study. Breast.

[10] Selber JC, Baumann DP, Holsinger CF (2012). Robotic harvest of the latissimus dorsi muscle: laboratory and clinical experience. J Reconstr Microsurg.

[11] Missana MC, Pomel C (2007). Endoscopic latissimus dorsi flap harvesting. Am J Surg.

[12] Dejode M, Barranger E (2016). Endoscopic 3D latissimus dorsi flap harvesting for immediate breast reconstruction. Gynecol Obstet Fertil.

[13] Iglesias M, Gonzalez-Chapa DR (2013). Endoscopic latissimus dorsi muscle flap for breast reconstruction after skin-sparing total mastectomy: report of 14 cases. Aesthet Plast Surg.

[14] Xu S, Tang P, Chen X, Yang X, Pan Q, Gui Y, Chen L (2016). Novel technique for laparoscopic harvesting of latissimus dorsi flap with prosthesis implantation for breast reconstruction: A preliminary study with 2 case reports. Medicine (Baltimore).

[15] Nakajima H, Fujiwara I, Mizuta N, Sakaguchi K, Ohashi M, Nishiyama A (2010). Clinical outcomes of video-assisted skin-sparing partial mastectomy for breast cancer and immediate reconstruction with latissimus dorsi muscle flap as breast-conserving therapy. World J Surg.

[16] Chung JH, You HJ, Kim HS, Lee BI, Park SH, Yoon ES (2015). A novel technique for robot assisted latissimus dorsi flap harvest. J Plast Reconstr Aesthet Surg.

[17] Clemens MW, Kronowitz S, Selber JC (2014). Robotic-assisted latissimus dorsi harvest in delayed-immediate breast reconstruction. Semin Plast Surg.

[18] Yuan H, Xie D, Xiao X, Huang X (2017). The clinical application of mastectomy with single incision followed by immediate laparoscopic-assisted breast reconstruction with latissimus dorsi muscle flap. Surg Innov.

[19] Lambaudie E, Houvenaeghel G, Walz J, Bannier M, Buttarelli M, Gurriet B (2008). Robot-assisted laparoscopy in gynecologic oncology. Surg Endosc.

[20] Lambaudie E, Narducci F, Leblanc E, Bannier M, Houvenaeghel G (2010). Robotically-assisted laparoscopic anterior pelvic exenteration for recurrent cervical cancer: report of three first cases. Gynecol Oncol.

[21] Narducci F, Collinet P, Merlot B, Lambaudie E, Boulanger L, Lefebvre-Kuntz D (2013). Benefit of robot-assisted laparoscopy in nerve-sparing radical hysterectomy: urinary morbidity in early cervical cancer. Surg Endosc.

[22] Hudry D, Ahmad S, Zanagnolo V, Narducci F, Fastrez M, Ponce J (2015). Robotically assisted para-aortic lymphadenectomy: surgical results: a cohort study of 487 patients. Int J Gynecol Cancer.

[23] Dindo D, Demartines N, Clavien PA (2004). Classification of surgical complications: a new proposal with evaluation in a cohort of 6336 patients and results of a survey. Ann Surg.

[24] Dauplat J, Kwiatkowski F, Rouanet P, Delay E, Clough K, Verhaeghe JL (2017). Quality of life after mastectomy with or without immediate breast reconstruction. Br J Surg.

[25] Bailey CR, Ogbuagu O, Baltodano PA, Simjee UF, Manahan MA, Cooney DS (2017). Quality-of-Life Outcomes Improve with Nipple-Sparing Mastectomy and Breast Reconstruction. Plast Reconstr Surg.

[26] Satteson ES, Brown BJ, Nahabedian MY (2017). Nipple-areolar complex reconstruction and patient satisfaction: a systematic review and meta-analysis. Gland Surg.

[27] Dent BL, Miller JA, Eden DJ, Swistel A, Talmor M (2017). Tumor-to-Nipple Distance as a Predictor of Nipple Involvement: Expanding the Inclusion Criteria for Nipple-Sparing Mastectomy. Plast Reconstr Surg.

[28] Petit JY, Veronesi U, Orecchia R, Rey P, Martella S, Didier F (2009). Nipple sparing mastectomy with nipple sparing areola intraoperative radiotherapy: one thousand and one cases of five years experience at the European Institute of Oncology of Milan (EIO). Breast Cancer Res Treat.

[29] Mallon P, Feron JG, Couturaud B, Fitoussi A, Lemasurier P, Guihard T (2013). The role of nipple-sparing mastectomy in breast cancer: a comprehensive review of the literature. Plast Reconstr Surg.

[30] Rusby JE, Smith BL, Gui GP (2010). Nipple-sparing mastectomy. Br J Surg.

[31] Murphy BL, Boughey JC, Hieken TJ (2017). Nipple-sparing mastectomy for the management of recurrent breast cancer. Clin Breast Cancer.

[32] Agresti R, Sandri M, Gennaro M, Bianchi G, Maugeri I, Rampa M (2017). Evaluation of local oncologic safety in nipple-areola complex-sparing mastectomy after primary chemotherapy: a propensity score-matched study. Clin Breast Cancer.

[33] Frey JD, Salibian AA, Choi M, Karp NS (2017). Mastectomy flap thickness and complications in nipple-sparing mastectomy: objective evaluation using magnetic resonance imaging. Plast Reconstr Surg Glob Open.

[34] Barrou J, Bannier M, Cohen M, Lambaudie E, Gonçalves A, Bertrand P (2017). Pathological complete response in invasive breast cancer treated by skin sparing mastectomy and immediate reconstruction following neoadjuvant chemotherapy and radiation therapy: Comparison between immunohistochemical subtypes. Breast.

[35] Lago V, Maisto V, Gimenez-Climent J, Vila J, Vazquez C, Estevan R. Nipple-sparing mastectomy as treatment for patients with ductal carcinoma in situ: a 10-year follow-up study. Breast J. 2018;24(3):298–303. 10.1111/tbj.12947 29139613

[36] Marino P, Houvenaeghel G, Narducci F, Boyer-Chammard A, Ferron G, Uzan C (2015). Cost-effectiveness of conventional vs robotic-assisted laparoscopy in gynecologic oncologic indications. Int J Gynecol Cancer.

